# Platelet activity in HIV-infected patients on abacavir-containing antiretroviral therapy

**DOI:** 10.1186/1758-2652-13-S4-P63

**Published:** 2010-11-08

**Authors:** R Palacios, JA González-Correa, J Ruiz, E Nuño, M Márquez, JP de la Cruz, J Santos

**Affiliations:** 1 Hosp. Virgen de la Victoria, Infectious Diseases Unit, Málaga, Spain; 2 Hosp. Virgen de la Victoria, Departamento de Farmacología, Málaga, Spain

## Background

Recent studies have shown an increase in the incidence of coronary heart disease in HIV-infected patients on treatment with abacavir (ABC), and platelet hyper-reactivity induced by this drug has been suggested as a possible cause.

## Objective

To assess platelet activity in HIV-infected patients with and without antiretroviral therapy (ART), analysing the influence of the presence or absence of ABC in the ART regimen.

## Patients and methods

Observational, cross-sectional, pilot study. Among HIV-infected patients on regular follow-up in our Centre, we selected 30 asymptomatic patients: 20 on ART for at least 24 weeks and with undetectable HIV viral load, 10 on ABC, and 10 on tenofovir (TDF), and 10 naïve patients, in addition to a control group of 10 HIV-negative subjects from the same hospital area. No subject was receiving drugs with antiagregant activity. Platelet activity was assessed by measuring time-dependent platelet aggregometry (electrical impedance on fasting whole blood), induced by ADP (1,25 y 2,5 µM), collagen (0.5 y 1 µg/mL), arachidonic acid (100 y 200 µM), and U46619 (receptor agonist of the tromboxano A_2_) (1.25 y 2.5 µM). A bivariate analysis by t student, anova and chi-square, and multivariate by linear regression were performed. Statistic program: SPSS, 16.0.

## Results

Demographic and anthropometric data, prevalence of cardiovascular risk factors, lipid profile and fasting glycemia were similar in all groups, but older age and longer time of HIV infection in the ABC group (50.4 vs 36.1, 34.2 and 42.7 years, respectively; p<0.05, and 140.3 vs 88.1 and 48.3 months in the two other groups of HIV patients; p< 0.05). Mean CD4 cells count in HIV-patients was 564/mm^3^. Platelet aggregation with exposure to U46619 was higher in the ABC group compared with the TDF group (11.1 vs 4.4%; p=0.007), naïve patients (11.1 vs 5.7%; p=0.014), and the HIV-negative group (11.1 vs 6.5%; p=0.04). These differences remained significant when controlled for age and time of HIV infection.

## Conclusions

ABC increases platelet aggregability possibly in relation with the receptor of tromboxano. Wider studies are needed to confirm this hypothesis.

